# PD-1 mediates functional exhaustion of activated NK cells in patients with Kaposi sarcoma

**DOI:** 10.18632/oncotarget.12150

**Published:** 2016-09-20

**Authors:** Asma Beldi-Ferchiou, Marion Lambert, Stéphanie Dogniaux, Frédéric Vély, Eric Vivier, Daniel Olive, Stéphanie Dupuy, Frank Levasseur, David Zucman, Céleste Lebbé, Damien Sène, Claire Hivroz, Sophie Caillat-Zucman

**Affiliations:** ^1^ Institut National de Recherche Médicale (INSERM) UMR1149, Centre de Recherche Sur l'Inflammation, Équipe Immunité Innée Chez l'enfant, Hôpital Robert Debré, Paris, France; ^2^ Université Paris Diderot, Sorbonne Paris Cité, Paris, France; ^3^ Institut Curie, Centre de Recherche, PSL Research University, INSERM U932 Immunité et Cancer, Paris, France; ^4^ Centre d'Immunologie de Marseille-Luminy, Aix-Marseille Université UM2, INSERM U1104, CNRS UMR7280, Marseille, France; ^5^ Immunologie, Hôpital de la Conception, Assistance Publique- Hôpitaux de Marseille, Marseille, France; ^6^ Centre de Cancérologie de Marseille, INSERM U1068, Equipe Immunité et Cancer, Institut Paoli-Calmettes, Aix-Marseille Université, CNRS, UMR7258, Marseille, France; ^7^ Hôpital Foch, Service de Médecine Interne, Suresnes, France; ^8^ Assistance Publique-Hôpitaux de Paris (AP-HP), Hôpital Saint-Louis, Département de Dermatologie, INSERM U976, Université Paris Diderot, Paris, France; ^9^ Assistance Publique-Hôpitaux de Paris (AP-HP), Hôpital Lariboisière, Département de Médecine Interne, Paris, France; ^10^ Assistance Publique-Hôpitaux de Paris (AP-HP), Hôpital Saint-Louis, Laboratoire d'Immunologie, Paris, France; ^11^ Present address: Assistance Publique-Hôpitaux de Paris (AP-HP), Hôpital Henri Mondor, Laboratoire d'Immunologie, Créteil, France

**Keywords:** NK cells, Kaposi sarcoma, PD-1, immune checkpoint, tumor escape

## Abstract

Programmed Death-1 (PD-1), an inhibitory receptor expressed by activated lymphocytes, is involved in regulating T- and B-cell responses. PD-1 and its ligands are exploited by a variety of cancers to facilitate tumor escape through PD-1-mediated functional exhaustion of effector T cells. Here, we report that PD-1 is upregulated on Natural Killer (NK) cells from patients with Kaposi sarcoma (KS). PD-1 was expressed in a sub-population of activated, mature CD56^dim^CD16^pos^ NK cells with otherwise normal expression of NK surface receptors. PD-1^pos^ NK cells from KS patients were hyporesponsive *ex vivo* following direct triggering of NKp30, NKp46 or CD16 activating receptors, or short stimulation with NK cell targets. PD-1^pos^ NK cells failed to degranulate and release IFNγ, but exogenous IL-2 or IL-15 restored this defect. That PD-1 contributed to NK cell functional impairment and was not simply a marker of dysfunctional NK cells was confirmed in PD-1-transduced NKL cells. *In vitro*, PD-1 was induced at the surface of healthy control NK cells upon prolonged contact with cells expressing activating ligands, i.e. a condition mimicking persistent stimulation by tumor cells. Thus, PD-1 appears to plays a critical role in mediating NK cell exhaustion. The existence of this negative checkpoint fine-tuning NK activation highlights the possibility that manipulation of the PD-1 pathway may be a strategy for circumventing tumor escape not only from the T cell-, but also the NK-cell mediated immune surveillance.

## INTRODUCTION

Natural Killer (NK) cells are cytotoxic innate lymphoid cells that are involved in the elimination of infected cells and tumors. Reactivity of NK cells toward target cells is tightly regulated by the integration of signals from activating and inhibitory cell surface receptors, which ultimately determine the magnitude of NK cell-mediated cytotoxicity and cytokine production [1–4]. NK cell activating receptors recognize stress-induced molecules at the surface of target cells, while inhibitory receptors mostly recognize major histocompatibility complex (MHC) class I molecules that contribute to host tolerance of NK cells. Target cells with low or no expression of MHC class I molecules do not provide inhibitory signals and therefore, become highly sensitive to NK cell-mediated elimination. The potential of NK cells to attack self cells is limited by tolerance mechanisms that set the triggering threshold of individual NK cells in order to prevent reactivity against self [5–7]. Moreover, tumors and infected cells develop various escape strategies to bypass NK-cell mediated surveillance [8–10].

PD-1 is a key immune checkpoint receptor expressed by activated T, B, myeloid and NKT cells [11–16], which negatively regulates antigen receptor signaling as part of the normal autoregulatory machinery. There are two PD-1 ligands that differ in their expression pattern. PD-L2 (B7-DC) is predominantly expressed on antigen presenting cells (APCs), whereas PD-L1 (B7-H1) is expressed in many lymphoid and non-lymphoid cell types and tumors [11, 17–19]. PD-L1 is further upregulated on lymphocytes and dendritic cells in response to cytokines. In T cells, binding of PD-1 to one of its ligands induces the dephosphorylation of signaling intermediates downstream of the T cell receptor (TCR), leading to functional inhibition [19–24]. Upon chronic antigen exposure, PD-1 is persistently upregulated on T cells. Consequently, PD-1 receptor/ligand interactions inhibit T cell activation, proliferation and cytokine production, and ultimately result in T lymphocyte exhaustion. Tumors and viruses have hijacked the PD-1/PD-L1 regulatory mechanism to avoid T cell-mediated surveillance of cancer or infected cells [25–29]. Most importantly, blockade of the PD-1 pathway results in reinvigoration of exhausted T cell-mediated responses, and therapies that target the PD-1/PD-L1 interactions have recently shown remarkable clinical responses in patients with various cancer types [30–33].

Kaposi sarcoma (KS) is a multifocal proliferation of lymphatic endothelium-derived spindle cells infected with the oncogenic virus, herpesvirus-8 (HHV-8) also called Kaposi-associated herpes virus (KSHV). Large leucocyte infiltrates are usually present in KS lesions, suggesting that active immune microenvironment is counterbalanced by immune inhibitory signals that prevent tumor elimination. We previously reported substantial alterations of NK cell functions in patients with KS, and identified distinct immunoevasion mechanisms that allowed HHV8 to escape NK cell responses and promote tumor cell growth [34]. Expression of PD-1 on NK cells was reported in different clinical settings [35–40], but its consequences on NK cell functions in tumor patients have not been evaluated in depth. Here, we show that PD-1 is expressed in a fraction of mature CD56^dim^ NK cells and associated with strongly altered functional capacities in KS patients. We demonstrate that PD-1 is induced *in vitro* on control NK cells upon sustained stimulation through activating ligands, and mediates inhibition of NK-cell degranulation and cytokine production. These data thus show that, as in T cells, expression of PD-1 on NK cells induces functional exhaustion, and support PD-1 as an immune checkpoint that controls NK cell activation upon chronic stimulation. An important implication of the present study is the possibility that therapeutic PD-1 blockade may be a strategy for circumventing tumor escape not only from the T cell-mediated, but also the NK cell-mediated immune surveillance.

## RESULTS

### PD-1 is expressed on a fraction of CD56^dim^ NK cells in KS patients

**Figure 1:**
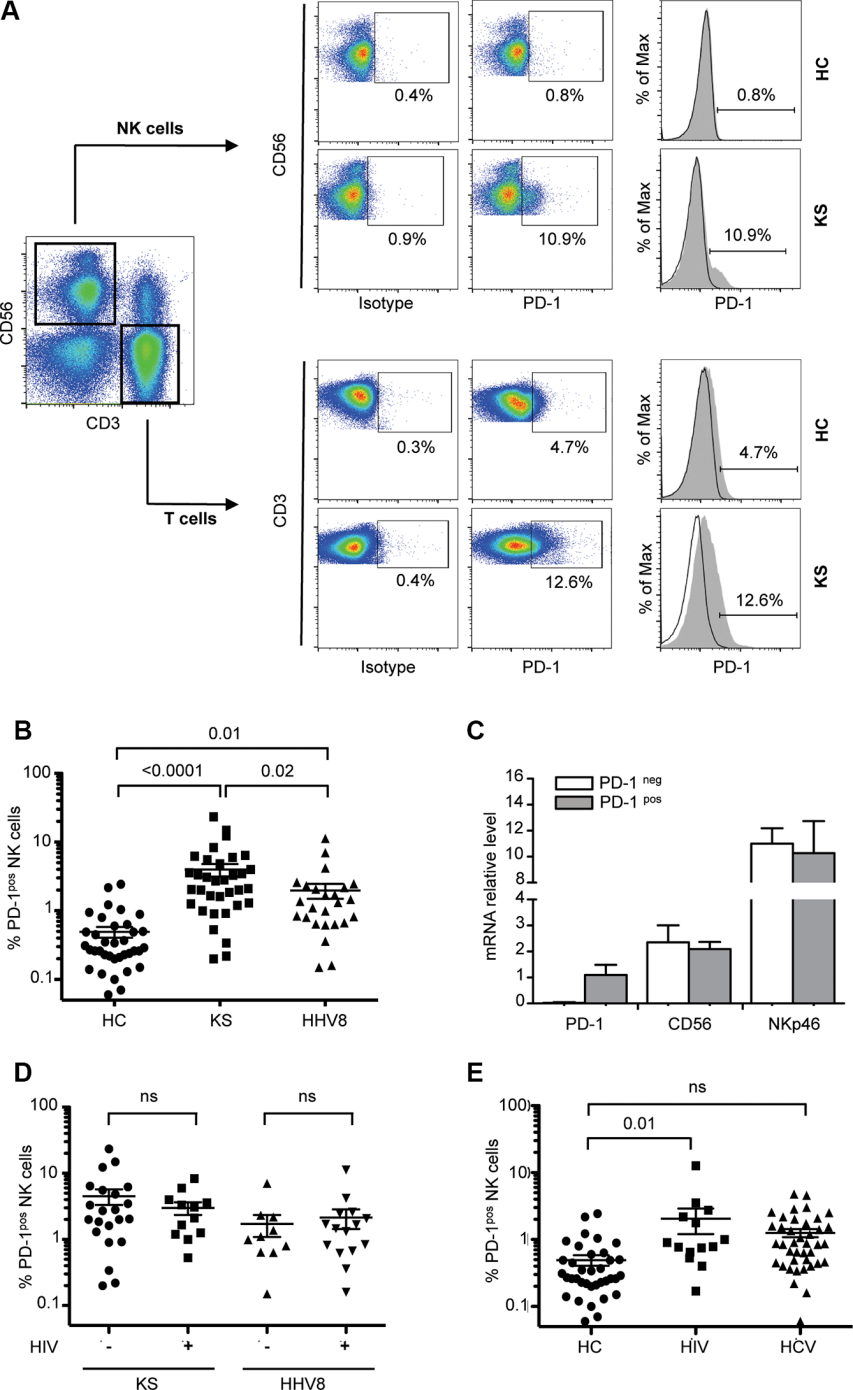
PD-1 is expressed on a fraction of CD56dim NK cells in KS patients NK cells were gated as follows: singlets, lymphocytes, CD3-CD56+ NK cells, and 7AAD- (live cells). Cells stained with FITC-labeled IgG control were used to establish the threshold for identifying PD-1^pos^ cells. (**A**) Representative dot plots (left panels) and histograms (right panels) showing *ex vivo* PD-1 staining on CD56^+^ NK cells in one patient with Kaposi sarcoma (KS) and one healthy control (HC). PD-1 staining on CD3 T cells from the same individuals is shown for comparison. (**B**) Statistical dot plots showing the percentage of PD-1^pos^ NK cells and corresponding mean ± SEM values (horizontal bars) in healthy controls (HC, *n* = 36), KS patients (KS+, *n* = 34) and HHV8 asymptomatic carriers (HHV8+, *n* = 25). *P* values were obtained by one-way ANOVA, followed by Tukey's multiple comparison test. (**C**) Summary graph showing mRNA levels of PD-1, CD56 and NKp46 relative to HPRT mRNA, in FACSAria sorted PD-1^pos^ (gray bars) and PD-1^neg^ (empty bars) NK cell subsets from 4 patients. (**D**) Percentage of PD-1^pos^ NK cells in KS patients and HHV8 asymptomatic carriers according to the presence or absence of HIV co-infection. (**E**) Percentage of PD-1^pos^ NK cells in HHV8-negative, ART-treated aviremic HIV+ patients (HIV+, *n* = 14), and in chronically infected HCV patients (HCV+, *n* = 41).

We found that a subset of NK cells from KS patients expressed PD-1 (mean frequency, 4.0% ± SEM 0.8% of NK cells vs. 0.5% ± 0.08% in age-matched healthy controls, *P* < 0.0001) (Figure 1A, 1B). PD-1^pos^ cells were exclusively detected among the CD56^dim^ population, and not in CD56^bright^ NK cells (Figure 1A). Elevated PD-1 levels were confirmed by qRT-PCR on sorted PD-1^pos^ versus PD-1^neg^ NK cells (Figure 1C).

To determine if the expression of PD-1 on NK cells was related to the HHV8-related tumor process or to the presence of HHV8 infection alone, we analyzed HHV8 asymptomatic carriers. We found PD-1^pos^ NK cells in HHV8 asymptomatic carriers, although at two times lower frequency than in KS patients (2.0% ± 0.5% of NK cells, *P* = 0.01 compared to healthy controls; *P* = 0.02 compared to KS patients) (Figure 1B). Since HHV8 infection frequently occurs in the context of HIV co-infection, we subgrouped KS patients and HHV8 asymptomatic carriers according to the presence or absence of HIV co-infection (Table 1). Yet, it must be noted that all HIV-positive subjects in our study were HIV-aviremic under antiretroviral treatment (ART). In both KS patients and HHV8 asymptomatic carriers, PD-1 expression was not different in HIV-positive and HIV-negative subjects (Figure 1D). We also analyzed a series of HHV8-negative, HIV-positive patients (ART-treated, HIV aviremic) and found PD-1^pos^ NK cells at a frequency comparable to that in HHV8 asymptomatic carriers (mean 2.1% ± 0.8%, *P* = 0.01 compared to healthy controls) (Figure 1E). Expression of PD-1 on CD56^bright^ NK cells was previously reported in patients with chronic hepatitis C [38]. We analyzed a series of HCV chronically infected patients and found a very small proportion of PD-1^pos^ NK cells (mean 1.3% ± 0.2%, *P* ns compared to controls) (Figure 1E). Like in HHV8- or HIV-positive subjects, all PD-1^pos^ NK cells from HCV-infected patients were found in the CD56^dim^ population, and not in CD56^bright^ NK cells. Moreover, PD-1 expression was not related to active HCV replication, as it was comparable in treatment-naïve patients and in patients with sustained response to IFNα/ribavirin-treatment (data not shown).

**Table 1:** Characteristics of the study subjects

	*n*	HIV viral load*	HCV viremia
**Kaposi Sarcoma**	34		
*HIV+*	12	undetectable	−
*HIV*−	22		−
**Asymptomatic HHV8**	25		
*HIV+*	15	undetectable	−
*HIV−*	10		−
**HIV+/HHV8−**	14	undetectable	−
**HCV+**	41		
*untreated*	*32*		+
*treated*	*9*		−
**Healthy controls**	36		−

* All HIV+ subjects were under efficient anti-retroviral treatment at time of study.

Notably, the proportion of PD-1^pos^ NK cells showed a considerable inter-individual heterogeneity, sometimes accounting for more than 20% of NK cells. This proportion was stable over the time, as observed by repeated analysis over 5-years follow-up in some patients (data not shown), indicating that the presence of PD-1^pos^ NK cells was not related to acute intercurrent events. No association was found between the frequency of PD-1^pos^ NK cells and age, gender, geographic origin, duration or severity of the underlying disease (active versus stable KS (data not shown). Expression of PD-1 on HIV-specific T cells was reported to positively correlate with plasma viral load in treatment naïve HIV-infected patients [41]. However, we found no correlation between PD-1 expression on NK cells and detectable HHV8 viremia, and observed only a weak correlation between the percentages of PD-1^pos^ NK cells and PD-1^pos^ CD8 T cells (*r* = 0.28, *P* = 0.01). Recently, PD-1^pos^ NK cells were observed in healthy individuals seropositive for cytomegalovirus [40]. However, we found no association between PD-1 expression on NK cells and the presence of CMV-specific IgG in patients (mean PD-1^pos^ NK cells, 4% in CMV-positive and 3.8% in CMV-negative patients, *p* = 0.65). Unfortunately, the CMV serological status of our healthy controls was not available. We also tested if expression of PD-1 on NK cells might be related to another blood-born persistent viral infection in which elevated proportions of PD-1^pos^ T cells have been described, such as Epstein Barr virus (EBV) infection. The frequency of PD-1^pos^ NK cells was not correlated with EBV viral load (*r* = −0.06, *P* = 0.8), making it our hypothesis unlikely. Elevated levels of circulating microbial products were shown to be responsible for the upregulation of PD-1 on monocytes in HIV patients [42]. Thus, we measured LPS and soluble CD14 levels in patients’ sera as indicators of microbial translocation, but found no correlation with PD-1 expression on NK cells (*r* = −0.15, *P* = 0.44 and *r* = 0.14, *P* = 0.42, respectively; data not shown).

Taken together, these results show that PD-1 was expressed in a fraction of CD56^dim^ CD16^+^ NK cells in KS patients, and to a lesser extent in patients with chronic viral infections (HHV8, HIV or HCV), without clear relationship between the proportion of PD-1^pos^ NK cells and clinical or biological characteristics of the underlying disease (duration, activity, response to treatment, viral load, associated infection).

### Expression of PD-1 on NK cells is associated with functional hyporesponsiveness

**Figure 2:**
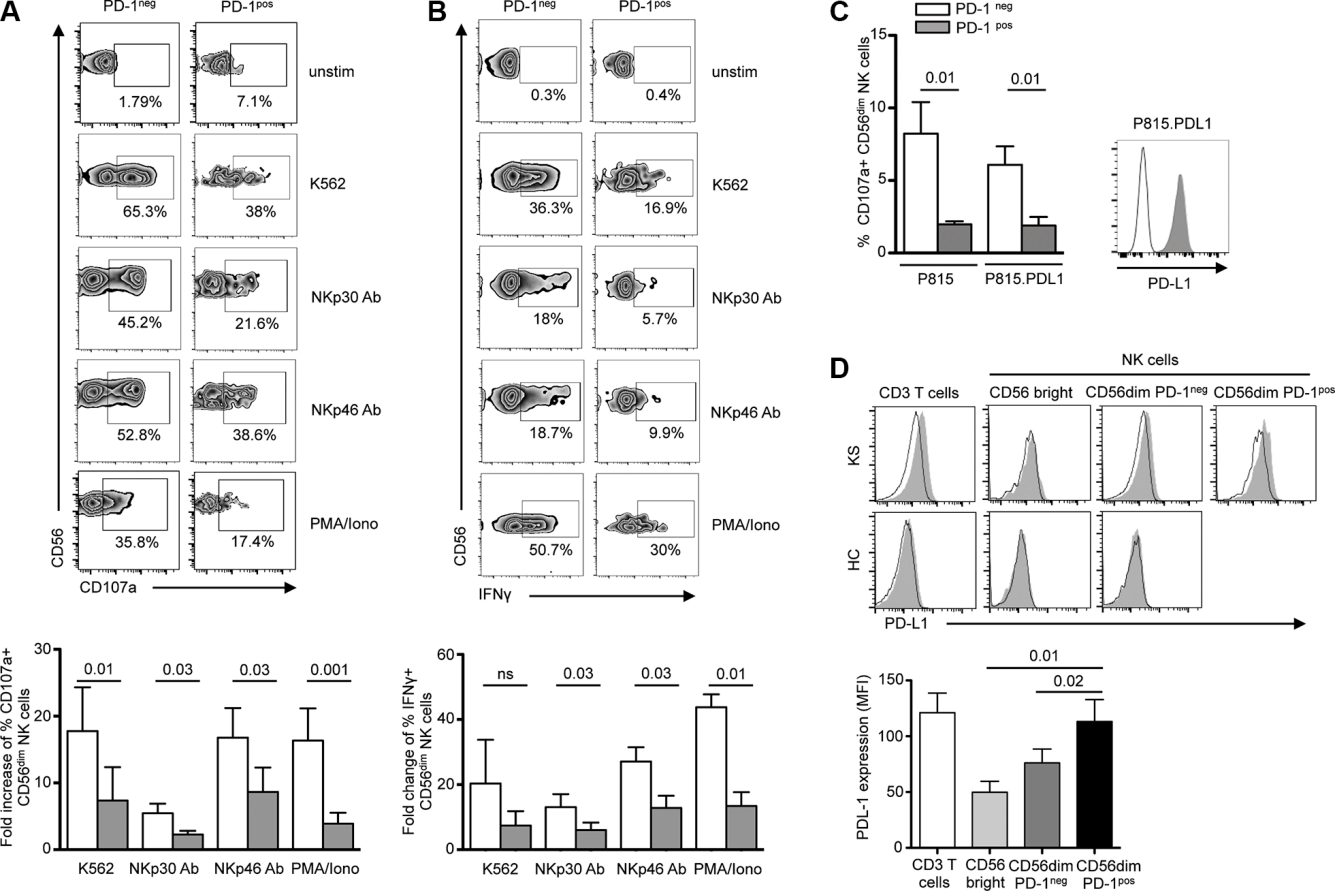
PD-1^pos^ NK cells are functionally hyporeactive (**A** and **B**) NK from KS patients were incubated for 5 hours in medium alone or in the presence of K562 target cells (effector:target 1:1), plate-bound anti-NKp30 or NKp46 mAb, 10 mg/ml each), or PMA plus ionomycin, after which CD107a degranulation (A) and IFNγ production (B) were compared in PD-1^pos^ and PD-1^neg^ NK cells. Representative zebra plots of surface CD107a and IFNγ expression in PD-1^neg^ and PD-1^pos^ NK cells (upper panels). Summary graphs showing fold-change of CD107a^+^ and IFNγ^+^ expression on PD-1^neg^ CD56^dim^ (empty columns) and PD-1^pos^ CD56^dim^ (gray columns) NK cells in the indicated condition relative to absence of stimulation (lower panels). *N* = 6 independent experiments. (**C**) NK cells from KS patients were stimulated for 5 hours with P815 or P815.PD-L1 target cells preincubated with 10 μg/ml anti-CD16 mAb or control isotype (effector:target ratio 1:1), after which CD107a expression was compared in PD-1^neg^ (empty columns) and PD-1^pos^ (gray columns) NK cells. Graphs show the summary of 6 independent experiments (left panel). A representative staining of PD-L1 expression on P815 (black line) and P815.PD-L1 (gray histogram) cells is shown (right panel). (**D**) *Ex vivo* PD-L1 staining on CD3 T cells, CD56bright and CD56dim NK cells from representative KS patient and healthy control. Control isotype is shown as thin black line (upper panel). Summary graph of PD-L1 expression (mean MFI) on T and NK cell subpopulations in KS patients (lower panel). Error bars indicate SEM. *P* values (Wilcoxon paired *t* test) are given.

Persistent expression of PD-1 on T cells renders them dysfunctional or exhausted. To assess the functional relevance of PD-1 expression on NK cells, we compared the *ex vivo* functional capacity of PD-1^pos^ and PD-1^neg^ NK cells from KS patients. Responsiveness was assayed by degranulation (cell surface expression of CD107a) and IFNγ production following 5-hour stimulation by HLA class I-negative K562 target cells, or direct triggering of NKp30 or NKp46 activating receptors with plate-bound agonist antibodies. Since PD-1 expression was confined to the CD56^dim^ subset of NK cells, comparison of PD-1^neg^ and PD-1^pos^ NK cells was restricted to the CD56^dim^ population to rule out functional differences between CD56^bright^ and CD56^dim^ NK cell subsets. At steady state, a fraction of PD-1^pos^ NK cells spontaneously expressed CD107a (5.9% of PD-1^pos^ vs 1.3% of PD-1^neg^ CD56^dim^ NK cells, *P* = 0.007). However, following stimulation, PD-1^pos^ NK cells responded much less efficiently than their PD-1^neg^ counterpart in all conditions of stimulation. This was reflected by a lower increase of CD107a expression after stimulation by K562 targets or by plate-bound NKp30 or NKp46 antibodies (Figure 2A). Similarly, IFNγ production was much lower in PD-1^pos^ than in PD-1^neg^ NK cells (Figure 2B). Even upon strong stimulation with PMA plus ionomycin, pharmacological activators that bypass early receptor signaling events, PD-1^pos^ NK cells responded poorly in both the degranulation and IFNγ production assays. Altogether, these results indicate that PD-1 expression on NK cells is associated with their functional hyporesponsiveness.

### PD-1 expression inhibits NK-cell effector functions

Binding of PD-1 to one of its ligands results in T lymphocyte functional inhibition. To more directly evaluate the involvement of PD-1/PD-1 ligand interactions in NK functional defect, we analyzed CD107a expression on PD-1^neg^ and PD-1^pos^ CD56^dim^ NK cells from KS patients using redirected degranulation assays with anti-CD16 antibody bound to P815 or P815.PD-L1 cells (Figure 2C). As expected, PD-1^neg^ cells strongly degranulated following CD16 triggering, and this response was comparable whatever P815 cells expressed PD-L1 or not. Similar to what was observed with plate-bound NKp30 or NKp46 antibodies, PD-1^pos^ NK cells were hyporesponsive to stimulation with CD16 antibody-coated P815 cells. However, the presence of PD-L1 on P815 cells did not further inhibit PD-1^pos^ NK cell degranulation. We thus wondered if PD-L1 expressed on NK cells themselves or on other mononuclear cells might be a source of ligands for PD-1^pos^ NK cells. In healthy controls, PD-L1 was not observed on peripheral T or NK cells. Conversely in KS patients, PD-L1 was detected on T cells and PD-1^pos^ CD56^dim^ NK cells, but not on CD56^bright^ or PD-1^neg^ CD56^dim^ NK cells (Figure 2D). This suggests that PD-1/PD-L1 homotypic (NK cell-NK cell) or heterotypic (NK cell-T cell) cell interactions may occur and inhibit NK cell effector functions, in agreement with previous observations in T cells [43, 44].

**Figure 3:**
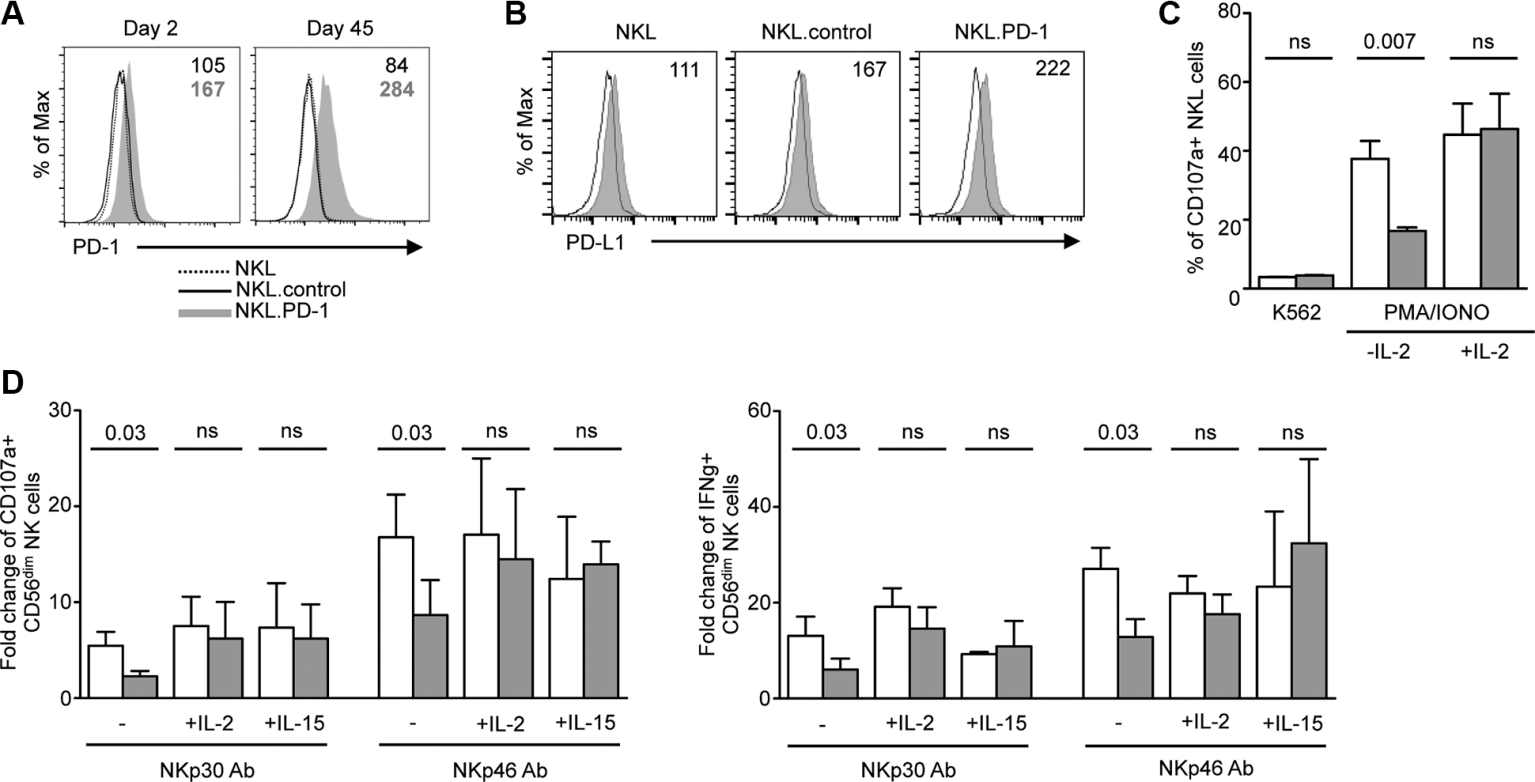
NK-cell hyporesponsiveness is directly related to PD-1 expression and is rescued by cytokines (**A**) NKL cell line was stably transduced with PD-1 lentiviral vector (NKL.PD-1) or control lentiviral vector (NKL.control). PD-1 expression was stable up to 45 days after transduction in NKL cells. Values in the quadrants indicate the MFI of PD-1 expression (MFI PD-1 mAb − MFI isotype control) on NKL.control and NKL.PD-1 cells. (**B**) Expression of PD-L1 was measured by flow cytometry on NKL, NKL.control and NKL.PD-1 cells (gray histograms). Control isotype is indicated as thin black line. Histograms are representative of 2 independent experiments performed in each cell line. MFI values are shown in the quadrants. (**C**) NKL.control (empty bars) and NKL.PD-1 (gray bars) cells were cultured in the presence or absence of IL-2 prior to 5-hour stimulation with K562 target cells (effector:target 1:1) or PMA plus ionomycin, after which CD107a surface expression was determined. Graphs show the percentage of CD107a^+^ cells in the indicated condition and represent the summary of at least 4 independent experiments. (**D**) PBMCs from KS patients were incubated overnight in the presence or absence of IL-2 (100 U/ml) or IL-15 (50 ng/ml) before 5-hour stimulation by plate-bound NKp30 or NKp46 mAbs. CD107a degranulation (left panel) and IFNγ production (right panel) were compared in PD-1^neg^ (empty columns) and PD-1^pos^ (gray columns) CD56^dim^ NK cells. Mean ± SEM of 3 to 6 independent experiments. Wilcoxon or Mann-Whitney *t* tests for paired and unpaired groups, respectively. *P* values are indicated.

To better understand if the impaired functions of PD-1^pos^ NK cells resulted from the expression of PD-1 itself, or whether the two phenomena were indirectly linked, we transduced the NKL cell line with a PD-1 lentiviral construct. Stable PD-1 expression was obtained and maintained up to 45 days (Figure 3A). Expression of PD-1 on transduced NKL cells did not influence the overall expression of classical NK cell differentiation, maturation or activation markers (data not shown). A weak expression of PD-L1 was observed on NKL.PD-1 cells, but not significantly more than on NKL.control cells (Figure 3B). We next analyzed if PD-1 expression rendered NKL cells hyporesponsive, as observed above for PD-1^pos^ NK cells in KS patients. NKL cells were cultured in the absence of IL-2 for 24 h prior to 5-hour stimulation with K562 cells (1:1 effector:cell ratio), after which CD107 expression was assessed. K562 cell stimulation did not induce significant degranulation in either NKL.control or NKL.PD-1 cells, in agreement with the previously reported low cytotoxicity of NKL cells against K562 targets [45]. Following stimulation by PMA plus ionomycin however, CD107a expression was strongly decreased in NKL.PD-1 compared to NKL.control cells. Importantly, if NKL.PD-1 cells were cultured in the presence of 100 U/ml of IL-2 before stimulation with PMA plus ionomycin, CD107a levels were strictly comparable to those in NKL.control cells (Figure 3C).

We thus wondered if exogenous cytokines could also restore functional abilities of PD-1^pos^ NK cells from KS patients. NK cells were preincubated overnight in the absence or presence of 100 U/ml IL-2 or 50 ng/ml IL-15 prior to stimulation with plate-bound NKp30 or NKp46 antibodies, and processed as above. Both cytokines rescued the capacity of PD-1^pos^ NK cells to degranulate and produce IFNγ to levels comparable to those of PD-1^neg^ cells (Figure 3D).

Collectively, these results indicate that PD-1 expression directly contributes to NK cell functional impairment. However, functional abilities of PD-1^pos^ NK cells can be rescued by cytokine treatment.

### PD-1 is expressed on *in vivo* activated, mature NK cells

**Figure 4:**
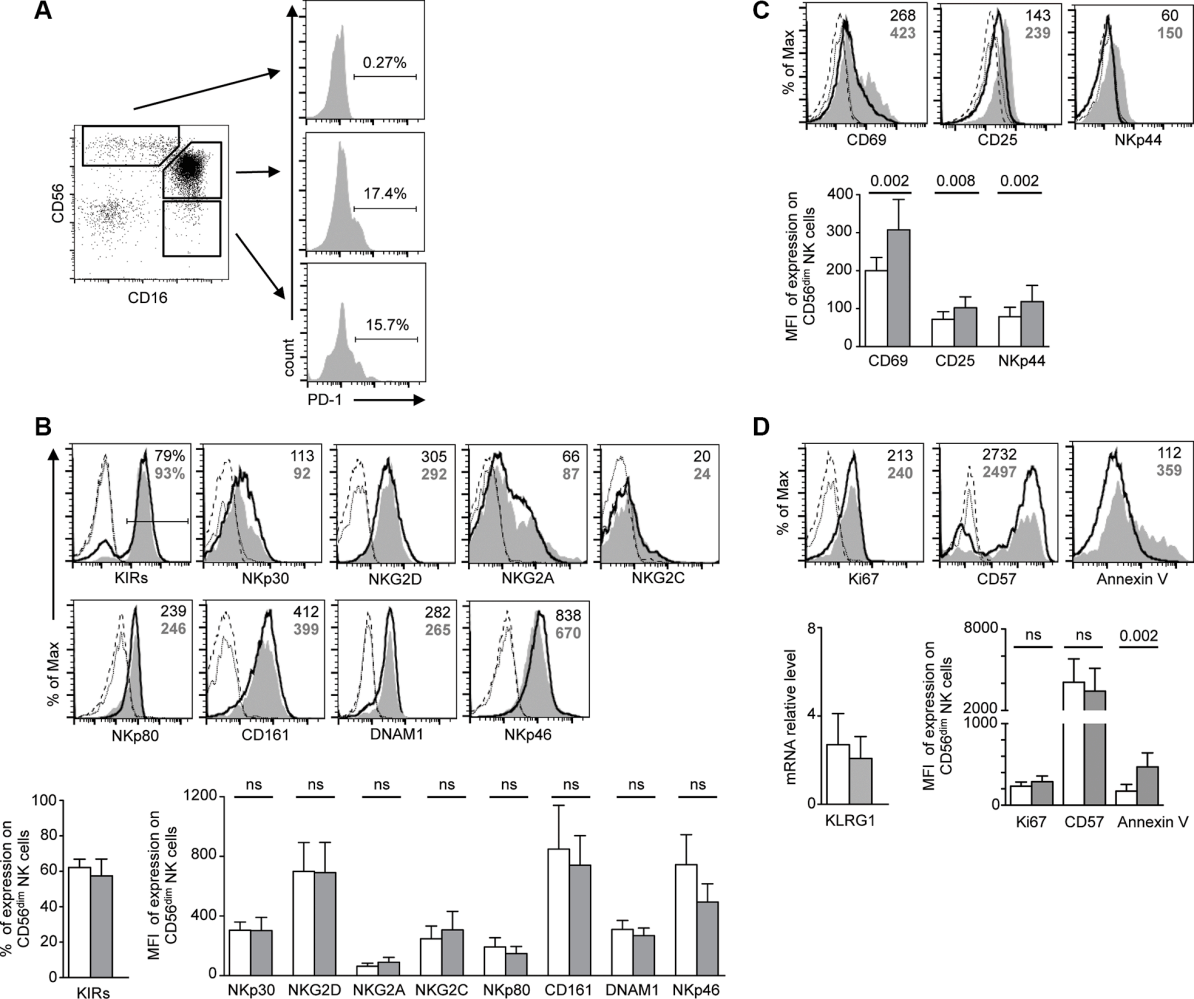
Phenotype analysis of PD-1^pos^ NK cells (**A**) PD-1 is expressed in the major CD56^dim^ CD16^+^ NK cell population and the very small fraction of CD56^−^ CD16^+^ NK cells, but is not expressed on CD56^bright^ CD16^−^ NK cells. Cells were gated on CD3− CD14− CD56+/− CD16+/− NK cells. (**B**–**D**) FACS analysis of the indicated activating and inhibitory NK cell receptors (B), activation markers (C) and proliferation or maturation markers (D). For each marker, representative staining of CD56^dim^ PD-1^pos^ (gray histograms) and CD56^dim^ PD-1^neg^ (empty histograms) NK cells, and control isotype on the respective population (dotted lines), are shown in the upper panels. KIR staining represents expression of whole KIRs, monitored by a cocktail of KIR antibodies. For KLRG1, mRNA levels relative to HPRT1 levels in sorted PD-1^pos^ and PD-1^neg^ NK cells are shown. Values in the quadrants indicate the percentage or the MFI of PD-1^pos^ (bold gray characters) and PD-1^neg^ (black characters) CD56^dim^ NK cells. Cumulative data for 9–12 patients are shown in the lower panel as mean ± SEM, analyzed by one-way ANOVA with Bonferroni's multiple comparisons test. *P* values are indicated.

NK cells are heterogeneous with respect to expression of several receptors often corresponding to NK maturation, differentiation and functional stages [46]. Expression of PD-1 was restricted to the mature CD56^dim^ CD16^+^ NK cell subset, and was never observed on the more immature CD56^bright^ CD16^−^ NK cell subset (Figure 4A). To determine if hyporesponsiveness of PD-1^pos^ NK cells was associated with a particular phenotype, we compared the expression patterns of a large panel of receptors in PD-1^pos^ and PD-1^neg^ CD56^dim^ NK cells from KS patients (Figure 4B). There were no significant differences in expression of HLA class I- specific inhibitory receptors (monitored by a cocktail of antibodies to Killer cell Immunoglobulin-like Receptors (KIR) KIR2DL1/S1, KIR2DL2/L3/S2, KIR3DL1/S1, KIR2DS4), or in expression of activating receptors such as NKp30, NKp46, NKG2D, NKG2A, NKG2C, NKp80, CD161 and DNAM-1.

PD-1 is expressed on activated T cells and functionally exhausted memory T cells. As shown above in degranulation assays, PD-1^pos^ NK cells from KS patients exhibited a basal expression of CD107a in the absence of *in vitro* stimulation (mean 5.2% in PD-1^pos^ vs. 2.0% in PD-1^neg^ NK cells, *P* = 0.007), suggesting that they were activated *in vivo*. Furthermore, PD-1^pos^ NK cells showed higher expression levels of the CD69 and CD25 activation markers than their PD-1^neg^ counterpart. They also expressed higher levels of NKp44, a natural cytotoxicity receptor (NCR) induced on NK cell surface upon activation (Figure 4C).

In mice, NK cells undergoing homeostatic proliferation after adoptive transfer become exhausted [47]. To determine if expression of activation markers in PD-1^pos^ NK cells mirrored recent *in vivo* proliferation, we analyzed NK cells from KS patients for the presence of proliferation and maturation markers (Figure 4D). Expression of Ki67, a nuclear protein associated with cell-cycle progression and generally used as a marker of recently divided cells, was not significantly different in PD-1^pos^ and PD-1^neg^ NK cells. At contrast with the NKG2A^−^KIR^+^CD57^+^ fully mature PD-1^pos^ NK cell population recently described in CMV-seropositive healthy controls [40], we found a similar proportion of PD-1^pos^ and PD-1^neg^ NK cells expressing CD57, a marker of CD56^dim^ late differentiation suggested to mark memory-like NK cells that have been expanded in response to infection [48]. We also analyzed expression of KLRG1, a marker of post-mature NK cells having undergone proliferation [49, 50]. Because commercial antibodies did not provide reliable KLRG1 staining on NK cells, we quantified KLRG1 mRNA levels in sorted PD-1^pos^ and PD-1^neg^ NK cells, and found no difference between the two populations. Finally, we observed that PD-1 expression was associated with a higher sensitivity of NK cells to spontaneous apoptosis, estimated by annexin V surface exposure (Figure 4D).

Altogether, our results indicate that PD-1 is expressed in a sub-population of NK cells showing markers of activation and apoptosis sensitivity, but is independent of their terminal maturation and proliferation state.

### PD-1 expression is induced by sustained stimulation via NK cell activating receptors

**Figure 5:**
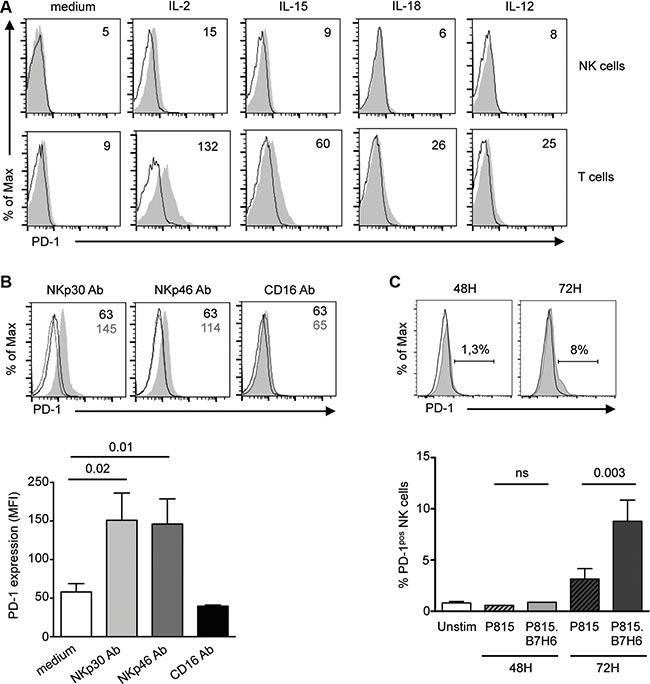
PD-1 is induced on control NK cells upon prolonged stimulation through the NKp30 or NKp46 activating receptors (**A**) PBMCs from healthy controls were left unstimulated or stimulated for 48 h with 100 U/ml IL-2, or 50 ng/ml IL-15, IL-18 or IL-12, and PD-1 expression (gray histograms) was monitored on CD3^−^CD56^+^ NK cells (upper panel). Control isotype is indicated as thin black line. Expression of PD-1 is shown on CD3^+^CD56^−^ T cells (lower panel) for comparison. Values in the quadrants indicate the MFI of PD-1 expression. Representative histograms of 3 independent experiments are shown. (**B**) Freshly purified control NK cells were stimulated for 72 h with 10 mg/ml of plate-bound antibodies to NKp30, NKp46 or CD16 (gray histograms) or their matching isotype control (black thick line) in the presence of IL-15 (50 ng/ml), after which PD-1 expression on CD56^dim^ NK cells was monitored by flow cytometry. PD-1 isotype control is shown as dotted lines. Representative histograms (upper panel) and summary graphs (lower panel) are shown. MFI of PD-1 expression are given in the quadrants. (**C**) Freshly purified control NK cells were left unstimulated or stimulated for 48 or 72 hours with P815 cells untransfected (black line) or transfected with the NKp30 ligand, B7-H6 (P815.B7-H6, gray histogram). Representative histograms (upper panel) and summary graphs (lower panel) are shown. Results represent the mean ± SEM of 4–6 independent experiments. *P* values are indicated.

The molecular mechanisms governing PD-1 expression are best defined in antitumor CD8 T cells. A link to persistent antigen exposure has been suggested [51]. Recently, soluble factors produced in the tumor microenvironment have also been involved in PD-1 induction [52]. We sought to understand the mechanisms contributing to PD-1 expression in NK cells. Healthy control PBMCs were cultured for 1 to 6 days with cytokines known to modulate NK cell proliferation, activation and/or effector functions, after which the cell surface expression of PD-1 was evaluated in parallel on NK and T cells. A very weak induction of PD-1 was observed following exposure to 100 U/ml IL-2, but at levels much lower than on T cells. IL-15, IL-12, IL-18, IFNα, IL-21, or IFNγ did not significantly induce PD-1 expression on NK cells (Figure 5A and data not shown).

The higher frequency of PD-1^pos^ NK cells in KS patients compared to asymptomatic HHV8 carriers suggested that factor(s) associated with tumor development or progression could be involved in the induction of PD-1 expression. We assessed expression of PD-1 following stimulation of healthy control NK cells with pro- or anti-inflammatory molecules, growth factors or angiogenic factors previously reported to play a role in KS pathogenesis, such as IL-1β, IL-6, IL-10, TGFβ, VEGF or prostaglandin E2 (PGE2). None of these factors induced PD-1 expression on NK cells (data not shown). We also determined if pathogen-associated molecules might play a role in PD-1 induction, by stimulating control PBMCs with agonists to TLR3 (poly(I:C)), TLR4 (LPS), TLR7/8 (R848) or TLR9 (CpG ODN 2006). No PD-1 induction was observed. Lastly, incubating control NK cells with the serum of KS patients exhibiting high frequency of PD-1^pos^ NK cells did not demonstrate any effect, suggesting that KS-associated soluble factors were not involved in PD-1 induction (data not shown).

Prolonged antigen-specific stimulation of the TCR maintains PD-1 expression on T cells. We wondered if sustained triggering of NK cell activating receptors could induce PD-1. We stimulated control NK cells for 72 hours with plate-bound antibodies to activating receptors or their respective isotype control, in the presence of IL-15 to maintain NK cell viability. As shown in Figure 5B, triggering of NKp30 or NKp46 reproducibly induced PD-1 expression on control NK cells. Importantly, induction of PD-1 upon stimulation through NKp30 or NKp46 did not extend to other activating receptors, as triggering with plate-bound CD16 antibody had no effect. Moreover, engagement of the NKG2D receptor by immobilized recombinant MICA molecules did not induce PD-1 (data not shown).

That sustained triggering of NKp30 could induce expression of PD-1 was confirmed using P815 cells stably transfected with the NKp30 ligand, B7-H6 [53]. As shown in Figure 5C, PD-1 was induced following 72 h stimulation of control NK cells with P815.B7-H6 but not with untransfected P815 cells, thus recapitulating the results with plate-bound NKp30 antibody. Taken together, these results indicate that sustained stimulation through the NKp30 or NKp46 activating receptors can lead to the induction of PD-1 expression on CD56^dim^ NK cells.

### Relationships between tumor cells and PD-1 expression on NK cells

**Figure 6:**
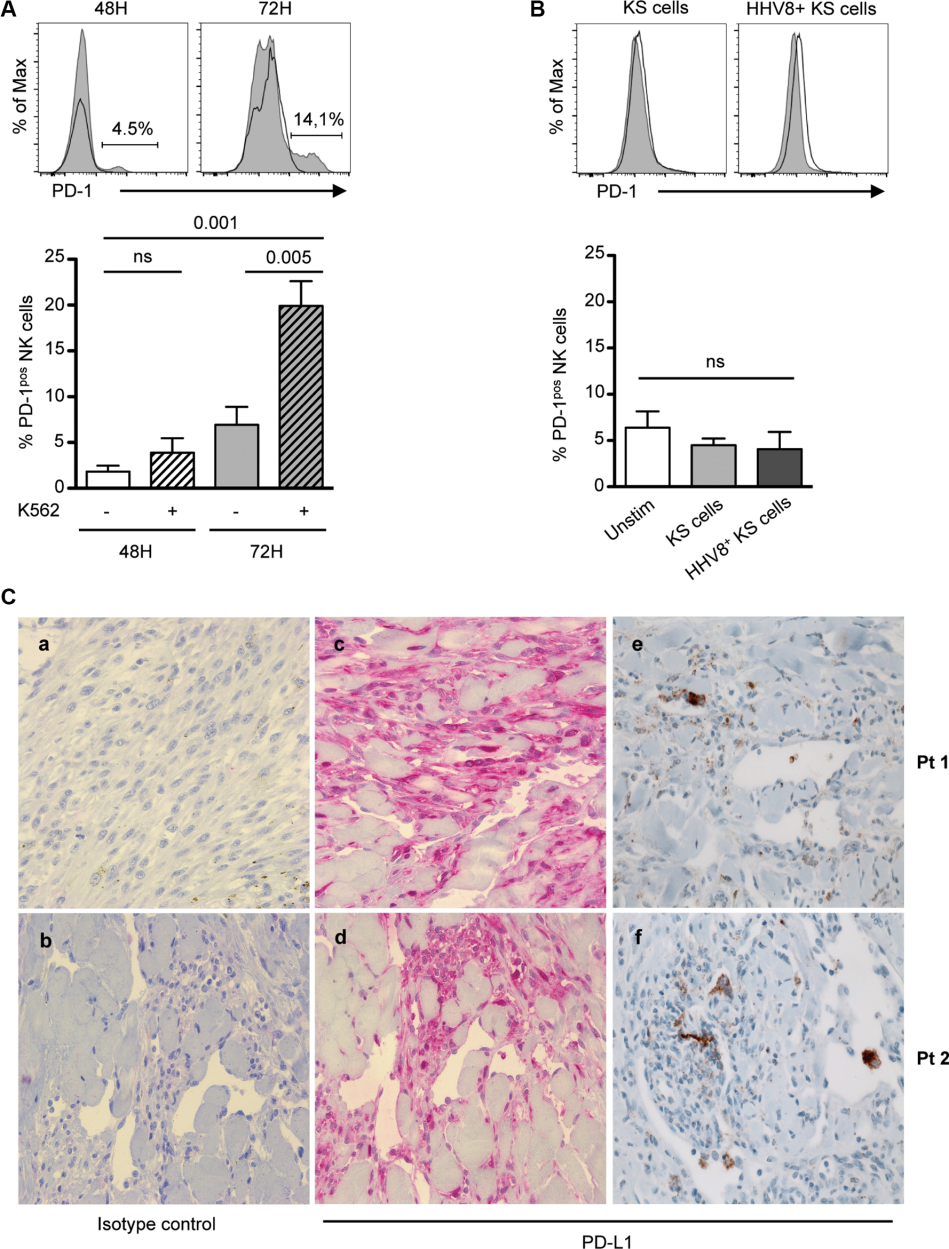
Relationships between tumor cells and NK cell PD-1 expression (**A** and **B**) Freshly purified control NK cells were left unstimulated (black line) or stimulated (gray histogram) for 48 or 72 hours with K562 cells (A), or with KS-derived or HHV8-infected KS-derived tumor cells (B), after which PD-1 expression on CD3^−^ CD56^dim^ NK cells was monitored by flow cytometry. Representative histograms (upper panels) and summary graphs (lower panel) are shown. Results represent the mean ± SEM of 4–6 independent experiments, analyzed by repeated measures ANOVA with Tukey's multiple comparisons. (**C**) Consecutive sections of paraffin-embedded KS biopsies were stained with anti-PD-L1 rabbit polyclonal (LS-B480) or monoclonal (E1L3N) primary antibody, or isotype control. Staining is detected with AP- (a–d) or HRP (e, f)- goat anti-rabbit IgG. Representative staining in 2 patients with classical KS, showing characteristic fascicles of spindle-shaped tumor cells and mononuclear infiltrates within the tumor microenvironment. Original magnification ×40.

Tumorigenesis can be accompanied by cell surface upregulation of NK cell activating ligands together with downregulation of MHC class I expression. The loss of MHC class I-specific inhibitory signals favors NK cell activation, and renders tumor cells sensitive to elimination by NK cells. Recent data in mice indicate that MHC class I-deficient tumor cells can escape from the immune response by inducing NK cell anergy, which can be reversed by exogenous cytokines [54]. We thus wondered if PD-1 was upregulated on NK cells following sustained stimulation cells expressing activating receptors in the absence of MHC class I inhibitory signals. Indeed, PD-1 was reproducibly induced on healthy control NK cells following 72 hours of stimulation with K562 cells, which express several ligands of activating receptors including B7-H6 [55] (Figure 6A). In HHV8-infected cells undergoing lytic replication, MHC class I expression is downregulated due to the effect of the viral K3 and K5 ubiquitin ligases expressed during the early lytic cycle [56, 57]. However HHV8 infection is predominantly latent within KS lesions [58]. As a model of HHV8-infected cells relevant to KS, we previously generated KS-derived cells persistently infected by a recombinant HHV8, which showed the same latency program as KS spindle cells [34]. Compared to uninfected cells, these HHV8-infected KS cells exhibited decreased but yet strong residual expression of MHC class I molecules, together with weak expression of NKp30 ligand [34]. We therefore, stimulated control NK cells up to 72 hours with HHV8-infected or uninfected KS-derived cells. No PD-1 induction was observed (Figure 6B), suggesting that PD-1 is best induced upon NK encounter with target cells expressing several activating ligands. Moreover, culturing NK cells with cell-free supernatants of HHV8-infected or uninfected KS cells did not demonstrate any effect (data not shown), indicating that soluble factors released by KS cells are likely not involved in PD-1 induction on NK cells.

Finally, we aimed to determine the potential relationship between PD-1^pos^ NK cells and PD-L1 expression within KS lesions. We previously reported that CD56-positive cells were very scarce in KS biopsy samples, suggesting that NK cells did not reach tumor lesions or could not survive in the tumor microenvironment [34]. As a consequence, it was not possible to detect intra-tumoral PD-1-positive NK cells. We therefore, analyzed PD-L1 expression within KS lesions (Figure 6C), and observed a focal staining of spindle-shaped tumor cells. Importantly, PD-L1 was predominantly expressed in inflammatory cell clusters in the tumor environment. This suggests that, like in other malignancies, PD-L1 expressed at the surface of KS tumor cells or inflammatory cells can mediate apoptosis of PD-1-positive cells.

## DISCUSSION

PD-1 has recently emerged as a key inhibitory checkpoint involved in T cell exhaustion and tumor escape [25–29]. Manipulation of the PD-1 pathway is an area of great interest as a strategy for circumventing tumor escape from T cell-mediated responses [30–33]. Here, we show that PD-1 may also represent an inhibitory checkpoint involved in NK cell exhaustion in patients with Kaposi sarcoma, and to a lower extent in patients with some chronic viral infections.

The presence of PD-1^pos^ NK cells was already reported in various clinical settings [35–40], although there were sometimes notable differences between the studies. In particular, PD-1 expression was reported on large expansions of CD56^bright^ NK cells in patients with chronic HCV infection, particularly in treatment non-responders [38]. At contrast, we found that PD-1 expression in HCV chronically infected patients was scarce, and always confined to the CD56^dim^ NK cell population, in agreement with others [35]. Whether such discrepancies are related to usage of distinct PD-1 mAbs or staining strategy remains to be determined. Indeed, different anti-human PD-1 clones have been shown to detect high and/or intermediate PD-1-positive cells [59], and the clone we used targeted cells with high rather than intermediate PD-1 expression. Large expansions of PD-1^pos^ NK cells were also reported in patients with renal cell carcinoma, multiple myeloma, EBV-associated lymphoproliferative disorder and ovarian carcinoma [35–37, 40]. Therefore, PD-1 may represent an inhibitory checkpoint expressed on NK cells in various cancers.

We found that PD-1^pos^ NK cells were hyporesponsive to short stimulation by activating NK cell receptors or by NK-cell sensitive K562 target cells. Importantly, this NK cell functional defect was not related to abnormal expression of activating or inhibitory receptors, was not associated with markers of proliferation or senescence, and was rescued by exogenous IL-2 or IL-15. Transducing PD-1 in NKL cells also induced their hyporesponsiveness. Thus, it is likely that PD-1 impedes NK-cell responses by directly inhibiting effector functions rather than by altering expression of other receptors. PD-1-mediated hyporesponsiveness may be due to alterations in intracellular signaling components, or to alterations in the spatial organization of membrane receptors, as described in mouse hyporesponsive NK cells [60]. Alternatively, PD-1 may act by upregulating genes in exhausted NK cells that impair their function. In HIV-specific CD8 T cells, PD-1 upregulated BATF, a transcription factor in the AP-1 family, and enforced expression of BATF was sufficient to impair T cell proliferation and cytokine secretion [61]. Whether PD-1 coordinately upregulates a program of genes in NK cells is currently under investigation by analyzing gene expression profiles from PD-1^pos^ cells.

We aimed at understanding the mechanisms involved in the chronic expression of PD-1 on NK cells. In mice, PD-1 can be induced on NK cells by various inflammatory factors including IL-2, IL-18, IFNγ TLR3 or TLR9 agonists [62]. Among several cytokines and inflammatory molecules tested, we found that only high dose IL-2 induced a very weak expression of PD-1 on control NK cells. PD-1 expression is also rapidly enhanced on mouse NK cells after acute infection by murine hepatitis virus strain-3 (MHV-3) or cytomegalovirus (MCMV) [63, 64]. In KS patients however, PD-1 expression on NK cells was stable over several years, making it unlikely that it was related to an acute phenomenon. Moreover, we found no evidence for viral reactivation in patients with PD-1^pos^ NK cell expansions and found no correlation with the CMV serological status, at contrast with the fully mature NKG2A^−^KIR^+^CD57^+^ PD-1^pos^ NK cell population recently described in CMV-seropositive healthy individuals [40]. None of the immunosuppressive soluble factors potentially produced in the tumor microenvironment induced expression of PD-1 on NK cells. In particular, we observed no effect of VEGF-A, a key angiogenesis-promoting factor secreted by Kaposi sarcoma and other tumor cells, and recently shown to enhance expression of PD-1 and other inhibitory checkpoints on intra-tumoral CD8 T cells [52].

The fact that PD-1^pos^ NK cells from KS patients exhibited some markers of activation *ex vivo* suggested us that PD-1 might be induced upon contact with (tumor) cells expressing a restricted set of activating ligands. Indeed, *in vitro* triggering of NKp30 or NKp46 receptors, but not of CD16 or NKG2D, significantly induced PD-1 on control NK cells. PD-1 was also expressed following sustained stimulation with murine cells overexpressing B7-H6, or with HLA class I-negative tumor cells expressing various activating ligands. Within KS lesions, HHV8 infection is predominantly latent and tumor cells express MHC class I molecules [34]. Indeed, *in vitro*-derived KS cell lines were unable to induce PD-1 on NK cells. It may be possible that only the small fraction of HHV8-infected cells that undergo reactivation of the lytic cycle, i.e. those exhibiting MHC class I loss together with expression of activating ligands, is able to induce PD-1 on NK cells. Whether such cells are present in low amount within the tumor and/or can be found outside the tumor, such as within tumor-draining lymph nodes or distant lymph nodes in HHV8-infected individuals remains to be determined.

Our results are reminiscent of a recent report in mice showing that NK cell anergy is induced as a result of persistent stimulation by MHC class I-deficient tumor cells in the absence of an activating inflammatory environment [54]. Whether mouse anergic NK cells expressed PD-1 was not determined. However, similar to PD-1^pos^ NK cells from KS patients, they were anergic to stimulation through cell surface activating receptors, but not to stimulation by cytokines. We therefore, propose that PD-1 represents a mediator of functional exhaustion in NK cells that have been subjected to prolonged exposure to cells expressing activating ligands, such as tumor cells. By analogy with CD8 cells, PD-1 would characterize a subset of NK cells that adapt to persistent stimulation by upregulating the PD-1 inhibitory receptor and turning off their responsiveness to stimulating conditions. PD-1 expression may serve to limit the activity of NK cells during antitumor immune response, thereby protecting tumor cells from NK-cell mediated elimination. Releasing the PD-1 immune checkpoint in NK cells might lead to NK-cell proliferation, intratumoral infiltration and increased effector functions, as shown for CD8 T cells [65].

## MATERIALS AND METHODS

### Subjects

KS patients and HHV8-infected individuals were described previously [34] (Table 1). Briefly, there were 34 patients with a history of KS (12 HIV-related and 22 classical KS) and 25 asymptomatic HHV8 carriers (15 HIV+ and 10 HIV- subjects). The HIV patient group consisted of 14 HHV8-negative ART-treated HIV patients. Importantly, all HIV+ patients in the different groups were aviremic upon antiretroviral treatment. The HCV patient group (ANRS HC-EP28 study) consisted of 41 chronically infected patients including 32 viremic (naive of treatment, or who discontinued treatment at least 6 months before study) and 9 aviremic subjects (sustained viral response following treatment). Healthy controls were 36 blood donor volunteers (HIV-, HCV- and HHV8-negative). The study was performed in accordance with the Declaration of Helsinki and French legislation, and received approval of the Saint-Louis and Pitié-Salpêtrière Hospital Ethical Committees (P040105 and A00822-37, respectively). All participants provided written informed consent.

### Flow cytometry

*Ex vivo* surface and intracellular staining was performed immediately after peripheral blood mononuclear cell (PBMC) isolation (Lymphoprep; Abcys). Cells were first incubated for 20 min at 4°C in the dark with FITC-conjugated or APC-conjugated anti-PD-1 antibody (MIH4, BD Biosciences, 1:50) or the respective isotype control. Cells were then washed and stained with a combinations of the following antibodies: Pacific Blue-conjugated anti-CD3 (UCHT1, 1:100); PE-conjugated anti-CD25 (M-A251, 1:25), anti-PD-L1 (MIH1, 1:10), anti-CD57 (NK-1, 1:25); APC-conjugated anti-CD161 (DX12, 1:25); FITC-conjugated anti-DNAM-1 (DX11, 1:10), anti-CD107a (H4A3, 1:10) (all from BD Biosciences); PE-Cy7-conjugated anti-CD56 (N901 (HLDA6), 1:50); PE-conjugated anti-CD69 (TP.55.3, 1:25), anti-NKp30 (Z25, 1:25), anti-NKp46 (BAB281, 1:25), anti-NKp44 (Z231, 1:25), anti-CD158e1e2 (KIR3DL1/S1, Z27.3.7, 1:25), anti-CD158i (KIR2DS4, FES172, 1:25); anti-CD158ah (KIR2DL1/S1, EB6.B, 1:25), anti-CD158bbj (KIR2DL2/L3/S2, GL183, 1:25), anti-CD16 (3G8, 1:25) (all from Beckman Coulter); PE-conjugated anti-NKG2D (1D11, eBioscience, 1:25); APC-conjugated anti-NKp80 (239127, 1:20), anti-NKG2A (131411, 1:20), anti-NKG2C (134591, 1:20) (all from R&D Systems). Dead cells were excluded with 7-Amino Actinomycin (7-AAD, Via-Probe, BD Biosciences). PE-Annexin V (BD Biosciences) staining was performed according to the manufacturer's instructions. For intracellular detection of Ki-67 and perforin, cells were fixed in 1% formaldehyde, permeabilized with 0.2% saponin and stained with PE-conjugated anti-Ki67 (B56, BD Biosciences, 1:10) or FITC-conjugated anti-perforin (dG9, BD Biosciences, 1:10). Samples were run using BD FACSCalibur flow cytometer in the first part of the study (quantification of PD-1^pos^ cells in patients and controls). Thereafter, all analysis was performed on BD LSR Fortessa. A total of at least 100,000 events in a live gate was collected. Gates were defined through isotype and fluorescence minus one (FMO) stains. The gating strategy was forward scatter versus side scatter, and NK cells were defined as CD3^−^ CD56+ CD16+/− lymphocytes. Data were analyzed using FlowJo software.

### Quantitative RT-PCR

NK cells were purified from PBMCs by negative selection using magnetic microbead separation technique (mean NK cell purity 97%, Miltenyi Biotec) and stained with FITC-conjugated anti-PD-1, Pacific Blue-conjugated anti-CD3, PE-Cy7-conjugated anti-CD56 and 7-AAD. PD-1^pos^ and PD-1^neg^ NK cells were then sorted on BD FACSAria II, and total RNA was extracted using RNeasy Mini kits (Qiagen) and retrotranscribed into cDNA using PrimeScript™ RT reagent Kit (Takara Bio Inc.). Expression of the following genes was analyzed with quantitative PCR using Light Cycler 480 detection system (Roche) and the following TaqMan real time ready single assays (Roche) according to the manufacturer's instructions: CD56 (assay identity: 111243), NKp46 (assay identity: 117489), PDCD1 (PD-1) (assay identity: 140931) and KLRG1 (assay identity: 126102). Gene expression was normalized to hypoxanthine phosphoribosyltransferase 1 (HPRT1, assay identity: 102079) mRNA levels.

### *Ex vivo* NK cell functional assays

PBMCs from patients exhibiting more than 3% of PD-1-positive NK cells were used for functional assays. In brief, PBMCs were incubated (5 × 10^5^ per U-bottom well) in the presence or the absence of K562 target cells (5 × 10^4^ per U-bottom well), plate-bound anti-NKp30 or anti-NKp46 mAbs (10 μg/ml, R&D Systems) or respective isotype control, or PMA (50 ng/ml) plus ionomycin (1 μg/ml) for 5 hr. FITC-conjugated anti-CD107a (BD Biosciences) was added directly at the beginning of the experiment. After 1 hr at 37°C in 5% CO2, brefeldin A (1 μg/ml) and monensin (6 μg/ml, Sigma) were added for additional 4 hrs. Cells were then harvested, washed, and stained with APC-conjugated anti-PD-1 antibody, followed by Pacific Blue-conjugated anti-CD3, PE-Cy7-conjugated anti-CD56, and 7-AAD. For intracellular IFNγ analysis, cells were fixed following surface staining, permeabilized with 0.2% saponin and stained with FITC-conjugated anti-IFN-γ antibody (BD Biosciences) for an additional 30 min. Where indicated, cells were preactivated overnight with IL-2 (100 U/ml) or IL-15 (50 ng/ml) before stimulation. For mAb-induced redirected degranulation experiments, PBMCs were incubated for 5 hours with untransfected P815 or P815.PD-L1 cells preincubated with anti-CD16 mAb (10 μg/ml, BD Biosciences) or control isotype (effector:target ratio 10:1), and processed as above.

### *In vitro* induction of PD-1 on control NK cells

PBMCs from healthy donors were stimulated in various conditions before evaluating PD-1 expression on CD56^+^ NK cells by flow cytometry. PBMCs were cultured for 1 to 6 days in RPMI 1640 medium supplemented with 10% heat-inactivated fetal calf serum (FCS) with different cytokines or growth factors: 100 U/ml IL-2, 50 ng/ml IL-15 or IL-12, 20 ng/ml IL-21, 10 ng/ml IL-1β (all from PeproTech); 50 ng/ml IL-18, 10 ng/ml IL-6, IL-10, IFNγ or TGFβ, 35 ng/ml VEGF (all from R&D Systems); or with TLR ligands: Poly(I:C) (TLR3 ligand, 5 μg/ml), CpG ODN 2006 (TLR9 ligand, 10 μg/ml), R848 (TLR7/8 ligand, 10 μg/ml) (all from Invivogen); or with prostaglandin E2 (PGE2, 100 ng/ml, Cayman Chemical).

Alternatively, freshly purified control NK cells were stimulated for 24–72 hours in flat-bottomed, high-protein-binding plates (Thermo Fisher Scientific) coated with 10 μg/ml of NKp30, NKp46, or CD16 antibodies or their respective isotype control, or 10 μg/ml of recombinant soluble MICA molecules [66], in the presence of IL-15 (50 ng/ml) to maintain NK cell viability.

K562 and P815 cells were cultured in DMEM supplemented with 10% fetal calf serum (FCS), 2% glutamine, and 1% penicillin-streptomycin. P815.PD-L1 and P815.B7-H6 transfected cell-medium contained G418 (0.5 mg/ml) in addition [53]. HHV8-infected KS-derived cells were cultured in MCDB131 medium supplemented with 10 ng/ml epidermal growth factor (EGF), 1 mg/ml hydrocortisone (Sigma), 10% FCS and G418 (0.7 mg/ml) as previously described [34]. Purified control NK cells were stimulated for 24–72 hours with the different cell lines (1:1 cell ratio) in the presence of IL-15, before evaluating PD-1 expression on CD56^+^ NK cells.

### PD-1 lentivirus generation and NKL cell transduction

Human PD-1 cDNA was PCR amplified from a pEYFP-N1-PD1 construct [67]. The PCR product was cloned into the lentiviral plasmid pDestDH1 containing the puromycin resistance gene using the Gateway technology (Invitrogen). The lentiviral plasmid pcDH1 containing the puromycin resistance gene was used as control vector (both plasmids were kindly provided by Philippe Benaroch and François-Xavier Gobert, Institut Curie, Paris, France). Replication-defective lentivirus particles pseudotyped with the envelope G protein of vesicular stomatitis virus G were produced in HEK 293T cells cotransfected with a mix of plasmids: psPAX2, pMD2.G (supplied by D. Trono; now Addgene plasmids 12260 and 12259, respectively) and either pDestDH1 PD-1 or pcDH1 plasmid. Supernatants were collected 48 h later and concentrated by ultracentrifugation (120,000 ×*g* for 90 min).

NKL cells, a human IL-2-dependent NK-cell line, were cultured in RPMI 1640 medium supplemented with 10% heat-inactivated FCS, 1% penicillin-streptomycin and 100 U/ml IL-2. For lentiviral infection, NKL cells were stimulated with IL-2 (1,000 UI/ml) and IL-21 (20 ng/ml) for 48 hours, washed and resuspended (2 × 10^6^/350 μl) in fresh medium supplemented with IL-2 and IL-21. Cells were plated onto flat-bottomed 12-well (2 × 10^6^ per well) and infected with 100 μl of concentrated virus supernatant containing protamine sulfate (8 μg/ml) and BX795 (6 μM, Invivogen). Following 4hr of incubation at 37°C in 5% CO2, supernatant was removed and cells were maintained in fresh medium supplemented with IL-2 (100 U/ml) for 24 hours. Cells were then washed and resuspended in fresh medium with IL-2 (100 U/ml) and puromycin (3 μg/ml). PD-1 expression was evaluated 48 hours later by FACS analysis.

For degranulation experiments, untransfected or transfected NKL cells were cultured in the absence of IL-2 for 24 h prior to stimulation with K562 target cells (effector:target 1:1) or PMA plus ionomycin, after which CD107a surface expression was determined.

### Immunohistochemistry

Following heat-induced antigen retrieval in pH 6.0 citrate buffer, formalin-fixed paraffin-embedded KS biopsy sections were incubated at 20°C for 1 hour with anti-PD-L1 rabbit polyclonal (LS-B480, LSBio) or monoclonal (E1L3N, Cell Signaling Technology) primary antibody, followed by incubation with alkaline phosphatase (AP)- or horseradish peroxidase (HRP)-linked goat anti-rabbit IgG, respectively.

### Statistical analysis

All statistical tests were performed with Prism software (GraphPad). Comparisons between two groups were performed using the Wilcoxon or Mann-Whitney *t* tests for paired and unpaired groups, respectively. Comparisons between more than two groups were performed using repeated measures or one-way ANOVA for paired and unpaired groups, respectively, followed by Tukey's multiple comparison test. Two-sided *p* values less than 0.05 were considered significant.
